# Recent Advances in the Vaccine Development Against Middle East Respiratory Syndrome-Coronavirus

**DOI:** 10.3389/fmicb.2019.01781

**Published:** 2019-08-02

**Authors:** Chean Yeah Yong, Hui Kian Ong, Swee Keong Yeap, Kok Lian Ho, Wen Siang Tan

**Affiliations:** ^1^Department of Microbiology, Faculty of Biotechnology and Biomolecular Sciences, Universiti Putra Malaysia, Serdang, Malaysia; ^2^Laboratory of Vaccines and Immunotherapeutics, Institute of Bioscience, Universiti Putra Malaysia, Serdang, Malaysia; ^3^Department of Pathology, Faculty of Medicine and Health Sciences, Universiti Putra Malaysia, Serdang, Malaysia; ^4^China ASEAN College of Marine Sciences, Xiamen University Malaysia, Sepang, Malaysia

**Keywords:** Middle East respiratory syndrome, coronavirus, animal model, vaccine, antibody dependent enhancement

## Abstract

Middle East respiratory syndrome (MERS) is a deadly viral respiratory disease caused by MERS-coronavirus (MERS-CoV) infection. To date, there is no specific treatment proven effective against this viral disease. In addition, no vaccine has been licensed to prevent MERS-CoV infection thus far. Therefore, our current review focuses on the most recent studies in search of an effective MERS vaccine. Overall, vaccine candidates against MERS-CoV are mainly based upon the viral spike (S) protein, due to its vital role in the viral infectivity, although several studies focused on other viral proteins such as the nucleocapsid (N) protein, envelope (E) protein, and non-structural protein 16 (NSP16) have also been reported. In general, the potential vaccine candidates can be classified into six types: viral vector-based vaccine, DNA vaccine, subunit vaccine, nanoparticle-based vaccine, inactivated-whole virus vaccine and live-attenuated vaccine, which are discussed in detail. Besides, the immune responses and potential antibody dependent enhancement of MERS-CoV infection are extensively reviewed. In addition, animal models used to study MERS-CoV and evaluate the vaccine candidates are discussed intensively.

## Introduction

Camel flu, or more commonly known as the Middle East respiratory syndrome (MERS), is a respiratory disease caused by MERS-coronavirus (MERS-CoV). MERS-CoV was first identified in Saudi Arabia in 2012 (Zaki et al., 2012). As of February 2019, 27 countries worldwide have reported cases of MERS-CoV infection, with 2,374 reported viral infection and 823 associated deaths, which corresponds to ∼35% fatality in identified cases (World Health Organization [WHO], 2019b), although the actual fatality rate of the viral infection is most likely below 35% due to some unidentified, mild, or asymptomatic cases. Majority of these cases occurred in Saudi Arabia, amounting to 1,983 of reported cases, with 745 associated deaths or ∼37.5% fatality (World Health Organization [WHO], 2019a).

Majority of the identified MERS-CoV cases are nosocomially acquired via direct close contact with infected patients (Chowell et al., 2015; Cauchemez et al., 2016), whereas cases of zoonotic transmission from dromedary camels to humans were reported primarily in Saudi Arabia, where human-camel interaction is frequent (Gossner et al., 2016). Hitherto, no specific treatments and vaccines are available for MERS-CoV infections. Although MERS-CoV is currently not listed as a potential pandemic threat, a recent outbreak in South Korea which demonstrated virus emergence in second and third generation contacts, has immediately raised concern that multiple mutations of MERS-CoV might cause enhanced human-to-human transmission (Wang et al., 2015b; Oh et al., 2018). Recently, MERS-CoV was added to the NIAID’s pathogen priority list as Category C Priority Pathogens due to its potential applications in biological warfare (Du et al., 2016b). Preventive measures against MERS-CoV infection, particularly vaccine development, are crucial to avoid deadly and unexpected future pandemics.

Middle East respiratory syndrome-coronavirus, the causative agent of MERS, is a positive sense, single-stranded RNA *Betacoronavirus* which belongs to the family of *Coronaviridae*. Its viral genome is about 30 kb in length, flanked by a 5’-terminal cap and 3’-poly(A) tail (van Boheemen et al., 2012; Scobey et al., 2013). MERS-CoV genome contains at least 10 open reading frames (ORFs), which encodes for 4 structural proteins: spike (S) protein, envelope (E) protein, membrane (M) protein, nucleocapsid (N) protein, 16 non-structural proteins (NSP1-NSP16), and 5 accessory proteins (ORF3, ORF4a, ORF4b, ORF5, and ORF8b) (van Boheemen et al., 2012; Du et al., 2017). Of all these viral proteins, S and N proteins are of particular interest in the development of vaccines against MERS-CoV, although other proteins such as E protein and NSP16 are potential immunogens as live attenuated vaccines (Almazan et al., 2013; Menachery et al., 2017).

## Criteria for an Effective Mers-CoV Vaccine

Two viral proteins of MERS-CoV, S and N proteins, were demonstrated to be highly immunogenic and capable of eliciting T-cell responses. However, only S protein was shown to induce neutralizing antibodies, the critical effectors against MERS-CoV (Agnihothram et al., 2014). Notably, N protein had also been proposed to be a potential protective immunogen for both neutralizing antibodies and T-cell immune responses through *in silico* approaches (Shi et al., 2015). Despite the prediction, no biological data have been presented thus far. Another potential B cell epitope of the MERS-CoV E protein was identified recently using *in silico* methods, yet similarly, no biological data were presented (Xie et al., 2018). Therefore, most of the MERS-CoV vaccine candidates are still based on the full length or part of the S protein.

Ideally, an effective MERS-CoV vaccine is required to induce both robust humoral and cell-mediated immunities, particularly antibody responses are crucial for the survival of the vaccinated hosts (Du et al., 2016b). Previous studies indicated that the level of serum neutralizing antibodies correlated positively with the reduction of lung pathogenesis, which increased the survival of animals challenged with MERS-CoV (Zhao et al., 2015; Zhang et al., 2016). In general, most of the potential MERS-CoV vaccine candidates were able to elicit systemic antibody responses, producing high titer of serum IgG upon immunization, but many failed to generate sufficient mucosal immunity unless the vaccines were administered via a mucosal or intranasal route. Activation of mucosal immunity is heavily dependent on the route of immunization, and this is a common challenge in vaccine development for many respiratory pathogens (Ma et al., 2014a; Guo et al., 2015). Pre-existing neutralizing mucosal antibodies are important as a first line of defenses against MERS-CoV infection (Guo et al., 2015). All neutralizing antibodies elicited by vaccines based on S protein could bind to the receptor binding domain (RBD) of the protein thereby inhibiting viral internalization and membrane fusion (Du et al., 2017). Little is known about the memory B-cell responses against MERS-CoV, apart from a recent study which demonstrated the persistence of anti-MERS-CoV antibodies in MERS survivors up to 34 months (Payne et al., 2016). On the other hand, antibody responses against another closely related coronavirus, SARS-CoV, were not persistent, whereby a 6-year follow-up study did not detect memory B-cell responses in SARS survivors (Tang et al., 2011). It is likely that some of the B-cells differentiate into MERS-CoV-specific memory B-cells following infection or vaccination, but the longevity and protective efficacy of these memory B-cells against MERS-CoV infection or re-challenge remain unresolved questions (Du et al., 2016b; Perlman and Vijay, 2016).

T-cell responses elicited by MERS-CoV vaccines also play important roles in protection against MERS. This is supported by the fact that viral clearance was impossible in T-cell deficient mice, but was possible in mice lacking B-cells (Zhao et al., 2014). Although T-cells are demonstrated to be a critical effector in acute viral clearance, protection for subsequent MERS-CoV infection is largely mediated by humoral immunity (Zhao et al., 2014). Several animal studies also demonstrated activation of T-cell responses following immunization with a MERS-CoV vaccine candidate, resulting in the elevated secretion of Th1 and Th2 cytokines (Lan et al., 2014; Ma et al., 2014a; Malczyk et al., 2015; Muthumani et al., 2015). It is also noteworthy to mention that adjuvants could be co-administered with MERS-CoV vaccines to tailor and possibly enhance the immune responses elicited by the vaccines. One study has indicated that co-administration of the MERS-CoV vaccine based on the S protein with Alum in mice resulted in a Th2 biased immunity, whereas a more robust Th1 and Th2 mixed immune response was produced when an additional adjuvant, cysteine-phosphate-guanine (CpG) oligodeoxynucleotides (ODN) was included in the formulation (Lan et al., 2014). To date, no detail investigation on MERS-CoV vaccine-induced memory T-cell responses is reported. However, MERS-CoV infection was shown to induce memory CD4+ and CD8+ T-cells responses in MERS survivors, at least up to 24 months (Zhao et al., 2017). There is little understanding about the biological function of memory CD4+ T-cells but they are likely to contribute to direct virus inhibition via cytokine production, particularly IFN-γ, and enhance the effector functions of CD8+ T-cells and B-cells (MacLeod et al., 2010). Although subsequent MERS-CoV infection is generally antibody mediated, memory CD8+ T cells are believed to facilitate virus clearance by eliminating infected cells (Kaech and Ahmed, 2001; Zhao et al., 2017). MERS survivors who later demonstrated strong virus-specific memory CD8+ T-cell responses were also shown to experience mitigated morbidity during the hospitalization period (Zhao et al., 2017). Similarly, the importance of T-cell responses against SARS-CoV was also highlighted in many studies (Channappanavar et al., 2014; Chu H. et al., 2014; Zhao et al., 2016). Interestingly, unlike SARS-CoV, MERS-CoV can infect both the CD4+ and CD8+ T cells in human, resulting in the downregulation of hDPP4, and induced intrinsic and extrinsic caspase-dependent apoptosis in T cells, which may lead to severe immunopathology (Chu et al., 2016). In addition, Chu et al. (2016) demonstrated the capability of MERS-CoV in infecting the T cells of common marmosets.

It is critical for a potential MERS-CoV vaccine to induce robust humoral and cell-mediated immunities. Although the protection against MERS-CoV is mainly mediated by humoral immunity, T-cell responses are crucial for acute viral clearance. Mucosal route is recommended for MERS-CoV vaccine delivery to induce the mucosal immunity in addition to the systemic responses. Persistence of the virus-specific antibodies induced by MERS-CoV vaccine is not thoroughly studied but represents a major challenge. An effective MERS-CoV vaccine is also required to induce immunological memory to provide a long-lived protection which in turn reduces the need of boosters, and in the long run will bring down the cost of vaccinations. Lastly, different adjuvants may also be used to improve the immunogenicity of MERS-CoV vaccines but would require detail studies on the interactions between them to ensure optimal vaccine efficacy and safety. So far, three potential MERS-CoV vaccines: a DNA vaccine and two viral vector-based vaccines have advanced into clinical trials (National Institutes of Health [NIH], 2016, 2018b, c).

## Potential Antibody Dependent Enhancement (ADE) of Mers-CoV Infection

Antibody dependent enhancement (ADE) is a condition whereby non-neutralizing antibodies are produced following an infection or a vaccination, which enhance the infectivity of the subsequent infection (Kuzmina et al., 2018). ADE of viral infections have been reported for dengue virus, human immunodeficiency virus, influenza virus, other alpha and flaviviruses, SARS-CoV, and Ebola virus (Dutry et al., 2011; Kuzmina et al., 2018). Thus, ADE is a critical issue that should be considered seriously in designing a MERS-CoV vaccine.

Attributed to the taxonomic and structural similarities between SARS-CoV and MERS-CoV, the processes involved in development of new vaccines against these two viruses, to a large extent, are similar. Vaccine candidates against SARS-CoV were initially developed based on the full-length S protein. However, these vaccines were later demonstrated to induce non-neutralizing antibodies which did not prevent MERS-CoV infection, and the immunized animals were not protected from the viral challenge instead they experienced adverse effects like enhanced hepatitis, increased morbidity, and stronger inflammatory responses (Weingartl et al., 2004; Czub et al., 2005). Many potential vaccines against MERS-CoV were also mainly focused on the same full-length S protein, raising a safety concern on the practical application of these vaccines (Du et al., 2016b).

To date, no ADE has been observed in MERS-CoV. Indeed, the ADE of SARS-CoV infection in human cells was only discovered 8 years after the virus was first identified in 2003 (Yip et al., 2011). Jaume et al. (2012) demonstrated that non-neutralizing antibodies induced by the full-length S protein of SARS-CoV facilitated the viral entry into host cells via a FcγR-dependent pathway. Our understanding about MERS-CoV is relatively lesser compared to SARS-CoV, mainly due to the fact that the former was discovered less than 7 years, thus it is unsurprising that the ADE of MERS-CoV has yet to be reported (Du et al., 2016b). Nevertheless, by employing appropriate strategies and methods, the ADE of MERS-CoV infection could be revealed in the future.

Two approaches have been suggested to mitigate the adverse effects of ADE. The first approach involves shielding the non-neutralizing epitopes of the S proteins by glycosylation, whereas the second approach, namely immunofocusing, aims to direct the adaptive immune responses to target only the critical neutralizing epitope to elicit a more robust protective immunity (Du et al., 2016a; Okba et al., 2017). A supporting evidence for the latter is that a MERS-CoV vaccine candidate based on a shorter S1 domain induced slightly stronger neutralizing activity than that based on the full-length S protein. In addition, a vaccine candidate based on the even shorter RBD induced the highest neutralizing immune responses (Okba et al., 2017).

## Current Animal Models Employed for Evaluation of Mers-CoV Vaccines

Animal models available for evaluation of MERS-CoV vaccines are highly limited, thus representing a huge challenge for vaccine development. MERS-CoV infects the human (Zaki et al., 2012), non-human primates-rhesus macaques (de Wit et al., 2013; Munster et al., 2013) and marmosets (Falzarano et al., 2014), and dromedary camels (Alagaili et al., 2014; Chu D.K. et al., 2014; Memish et al., 2014). The first animal model adopted for the development of MERS-CoV vaccine was rhesus macaques (de Wit et al., 2013; Munster et al., 2013). They demonstrated clinical symptoms of MERS-CoV infection including an increase in respiratory rate and body temperature, hunched posture, piloerection, cough, and reduced food intake. Radiographic imaging analysis also revealed varying degree of pulmonary diseases following infection. Although the viral RNA of MERS-CoV was detected in most of the respiratory tissues, but viral tropism was restricted primarily to the lower respiratory tract. Rhesus macaques infected with MERS-CoV experienced transient, mild to moderate disease severity (van Doremalen and Munster, 2015; Du et al., 2016b). It is noteworthy that the pathological changes induced in rhesus macaques infected by MERS-CoV were the results of the host inflammatory responses triggered by the virus instead of the direct viral cytolytic activity (Prescott et al., 2018).

The common marmoset is another frequently used animal model to evaluate MERS-CoV vaccines (Falzarano et al., 2014). Similar to rhesus macaques, humoral and cell-mediated immunities could be detected in these animals following MERS-CoV vaccination. The common marmosets infected with MERS-CoV developed moderate to severe acute pneumonia and increased viral load in the respiratory tract in addition to other clinical symptoms experienced by rhesus macaques (van Doremalen and Munster, 2015; Yu et al., 2017). Intriguingly, the common marmoset also demonstrated signs of renal damage as in human cases following MERS-CoV infection, and the viral RNA could be detected in other non-respiratory organs contrary to rhesus macaques (van Doremalen and Munster, 2015; Yeung et al., 2016). Falzarano et al. (2014) also reported that the common marmoset could serve as a partially lethal animal model. Similarly, Chan et al. (2015) demonstrated that marmosets challenged with MERS-CoV developed severe diseases, leading to fatality. Thereafter, marmosets have been successfully used as a moderate and severe model to study MERS-CoV (Baseler et al., 2016; Yeung et al., 2016; Chen et al., 2017; van Doremalen et al., 2017; Yu et al., 2017; de Wit et al., 2019).

The dromedary camels serve as a natural reservoir for MERS-CoV, and are responsible for zoonotic transmission of the virus to humans. Mild clinical symptoms such as increase in body temperature and rhinorrhea were observed in the dromedary camels infected with MERS-CoV (Adney et al., 2014). Interesting, MERS-CoV tropism in dromedary camels is limited to the upper respiratory tract, and is less apparent in the lower respiratory tract, contrary to rhesus macaques (Adney et al., 2014). The viral RNAs of MERS-CoV are detectable in the respiratory tract, lymph node and the excreted breath of the infected dromedary camels. Viral shedding from the upper respiratory tract of the dromedary camels may explain the efficiency of virus transmission among the camels, and from camels to humans (Adney et al., 2014). The dromedary camels immunized with MERSV-CoV vaccines were also shown to activate both the B-cell and T-cell responses (Muthumani et al., 2015; Haagmans et al., 2016; Adney et al., 2019).

Although camels are the natural reservoirs of MERS-CoV, whilst macaques and marmosets are closely related to the human, the handling of these large mammals is laborious and costly. The lack of small animal models for the initial screening of potential vaccine candidates greatly hampers the development of MERS-CoV vaccines. Unlike SARS-CoV, MERS-CoV does not readily infect smaller rodents such as mice or hamsters due to the substantial differences in the viral binding receptors, dipeptidyl peptidase 4 (DPP4) (Goldstein and Weiss, 2017). Nevertheless, considerable amount of efforts have been devoted to produce MERS-CoV-permissive small rodents for evaluation of MERS-CoV vaccines. Mice transduced by a viral vector to express human DPP4 (hDPP4) were shown to be susceptible to MERS-CoV infection, manifested by the development of pneumonia and histopathological changes in the lungs. However, viral clearance in these infected mice was observed at day-8 post-infection, failing to recapitulate severe human diseases (Zhao et al., 2014). Later, a more established transgenic mouse model expressing hDPP4 globally was developed, and it was the first lethal animal model available to evaluate MERS-CoV vaccines. Mortality was noted in these mice within days post-infection, and virus dissemination to other organs was observed with exceptionally high titer detected in the lung and brain (Agrawal et al., 2015). Recently, a transgenic mouse model was produced by replacing the full-length mouse *DPP4* gene with the human equivalent. However, these transgenic mice did not demonstrate any sign of diseases following the MERS-CoV infection, and no virus dissemination to other organs was observed (Pascal et al., 2015). CRISPR/Cas9 was also previously employed to sensitize the mice to MERS-CoV infection by substituting two amino acids at positions 288 and 230 of the mouse DPP4. Although these genetically engineered mice allowed viral replication in the lungs, they did not experience apparent morbidity following infection by the wild-type MERS-CoV. Severe diseases were observed only when the mice were infected by mouse-adapted MERS-CoV generated via 15 serial lung passages (Cockrell et al., 2016). As mouse DPP4 is vital to normal glucose homeostasis and immunity, altering the mouse DPP4 could have unforeseen complications to the mouse model (Fan et al., 2018). Therefore, another transgenic mouse model has been introduced, in which the *hDPP4* gene was inserted into the genome of C57BL/6-mouse at Rosa26 locus using the CRISPR/Cas9 technology. This mouse model, namely R26-hDPP4, when infected by MERS-CoV at low dose, developed severe lung diseases related to acute respiratory symptoms (ARDS) and central nervous system (CNS). In addition, the R26-hDPP4 is also susceptible to infection by a MERS-CoV pseudovirus, serving as an alternative to test MERS-CoV vaccines in the absence of BSL-3 facility (Fan et al., 2018). All of the animal models described above are summarized in Table 1.

**TABLE 1:** Animal models used for vaccine development against Middle East respiratory syndrome-coronavirus.

**Animal models**	**Results**	**Advantages/Limitations**	**References**
Rhesus macaques	The animals manifested clinical signs within 24 h following infection, including increase in respiratory rate and body temperature, hunched posture, piloerection, cough, reduced food intake and varying degree of pneumonia. No mortality was observed in the infected animals throughout the study. The increase in white blood cell counts was early and transient, and viral-load was reported to be higher in lower respiratory tract and decreased overtime.		Munster et al., 2013
Rhesus macaques	The animals experienced early increase in neutrophil at day-1 post-infection (P.I.), and restored at day-3 P.I. Development of pneumonia in the animals was rapid after the infection but short-lived. No mortality or virus dissemination to other non-respiratory tissue was observed in the infected animals. Infection is restricted primarily at lower respiratory tract.	–Genetically closer to human –Do not recapitulate severe diseases in human –Expensive model due to high husbandry requirement	de Wit et al., 2013
Rhesus macaques	The rectal temperature of the animals increased at 1 to 2 days P.I. and restored thereafter. Extensive lung lesions and varying degree of inflammation were observed in the lungs of the animals collected at day-3 P.I. Other pathological changes of the infected lungs include interstitial pneumonia, pulmonary edema, hemorrhaging, degeneration and necrosis of pneumocytes and bronchial epithelial cells. No sign of damage in other non-respiratory organs was observed.		Yu et al., 2017
Immunosuppressed rhesus macaques	The immunosuppressed animals developed rapid pneumonia but less severe than the non-immunosuppressed monkey. Higher viral load, viral shedding, and virus dissemination to other non-respiratory organs were observed in the immunosuppressed animals following infection.	–Potential model for mimicking MERS-CoV infection in immunocompromised patients –Expensive model due to high husbandry requirement –Additional treatment is required to produce immunosuppressed animals	Prescott et al., 2018
Common marmosets	Most of the infected animals developed progressive severe pneumonia characterized by interstitial infiltration. Some animals were euthanized because of diseases severity. Extensive lung lesions were observed in all the infected animals at different necropsies time points. Viral RNA could be detected in blood, respiratory organs and other non-respiratory organs including kidney, suggesting virus dissemination.	–Severe, partially lethal animal model –Able to manifest renal damage –Expensive model due to high husbandry requirement	Falzarano et al., 2014
Common marmosets	Infected animals developed severe pneumonia at day-3 P.I. characterized by exudative pathological changes with widespread pulmonary edema, hemorrhaging, and huge number of inflammatory cells.		Yu et al., 2017
Common marmosets	*In vitro* analysis using lung and kidney cells showed that hyperexpression of Smad7 or FGF2 induced by MERS-CoV led to an immense apoptotic response. Common marmosets infected with MERS-CoV demonstrated acute respiratory distress and disseminated infection in kidneys and other organs.		Yeung et al., 2016
Dromedary camels	The animals infected experimentally with MERS-CoV developed mild symptoms such as increase in body temperature and rhinorrhea. Symptoms of the infected animals lasted less than 2 weeks. Shedding of infectious virus was detected in less than 7 days P.I. but viral RNA remained detectable up to 35 days P.I. in the nasal swabs. Viral RNA, but not infectious virus, was detected in the exhaled breath of the infected animals at day-3 and -5 P.I. The Infection was restricted to upper-respiratory tract.	–Potential model for pathogenesis studies of MERS-CoV and transmission to human –Do not recapitulate severe diseases in human –Expensive model due to high husbandry requirement	Adney et al., 2014
hDPP4-transduced mice	Mice transduced with adenoviral vector to express hDPP4 in lungs were susceptible to MERS-CoV infection. Following the infection, mice developed interstitial pneumonia in addition to reduced weight gain in young mice and weight loss in aged mice. No mortality was observed in all infected animals, and virus clearance was detected at day-6 to -8 P.I. Expression of hDPP4 in the animals’ lungs lasted for 17 to 22 days after transduction.	–Ease of manipulation –Low husbandry requirement –Readily available methods in testing vaccine efficacy –Do not recapitulate severe diseases in human	Zhao et al., 2014
Transgenic mice expressing hDPP4 globally	Following the infection, the transgenic animals developed severe pneumonia, and 100% mortality was detected at day-6 P.I. Virus dissemination to other non-respiratory organs was detected with significantly high viral RNA in the brains and lungs. No viral RNA could be detected in the kidney or the liver of the infected mice.	–Lethal animal model –Ease of manipulation –Low husbandry requirement –Readily available methods in testing vaccine efficacy –Lack physiological expression pattern because all mouse cells express hDPP4	Agrawal et al., 2015
hDPP4-humanized transgenic mice	Humanized mice can be infected with MERS-CoV but do not demonstrate clinical sign of diseases. Pathological changes including peri-bronchiolar inflammation, interstitial infiltration, and minimal peri-vascular inflammation were observed at 2 to 4 days P.I. Viral RNA was detected in the lungs, and no virus dissemination to other organs was observed	–Ease of manipulation –Low husbandry requirement –Readily available methods in testing vaccine efficacy –Correct physiological expression pattern –Little to no clinical sign	Pascal et al., 2015
CRISPR/Cas9-engineered mice	Mice genome was modified to incorporate human codons at amino acid positions 288 and 330 in the mouse *DPP4* gene causing them to become susceptible to MERS-CoV infection. The infected mice did not demonstrate any sign of diseases but supported viral replication in the lungs. Inflammation of the infected lungs was moderate. Severe disease could be induced in these mice by infecting them with mouse-adapted MERS-CoV.	–Severe, partially lethal animal model (challenged with mouse-adapted MERS-CoV only) –Ease of manipulation –Low husbandry requirement –Readily available methods in testing vaccine efficacy	Cockrell et al., 2016
hDPP4-knockin mice using CRISPR/Cas9	hDPP4-knockin mice were susceptible to MERS-CoV infection. The mice experienced drastic weight loss above the typical euthanization endpoint (20%) by day-5 P.I. Lesions and virus load were detected in the brains and the lungs of the mice but not in the kidneys or livers.	–Lethal animal model –Ease of manipulation –Low husbandry requirement –Readily available methods in testing vaccine efficacy –Lack physiological expression pattern because all mouse cells express hDPP4	Fan et al., 2018

*MERS-CoV, Middle East respiratory syndrome-coronavirus; DPP4, dipeptidyl peptidase 4; hDPP4, human dipeptidyl peptidase 4; P.I., post-infection.*

Apart from the mouse model, rabbits were also reported to be asymptomatically infected by MERS-CoV. By extensive research, these animals could represent another potential animal model to evaluate MERS-CoV vaccines (Haagmans et al., 2015). Smaller animal models are more economically available to vaccine evaluations in addition to the ease of animal manipulation and readily available methods in testing vaccine efficacy.

## Current Mers-CoV Vaccine Platforms

As of now, SARS-CoV and MERS-CoV are the only coronaviruses known to cause severe diseases in human. Development of SARS vaccines was mainly focused on the S protein of SARS-CoV (Bukreyev et al., 2004; Weingartl et al., 2004; Yang et al., 2004; Czub et al., 2005; Kam et al., 2007; Lin et al., 2007; Fett et al., 2013). To date, no vaccine has been licensed to prevent MERS-CoV infection. Although several vaccine candidates are currently in clinical trials, many still remained in the pre-clinical stage. Current approaches for the development of MERS-CoV vaccines are mostly referred to the methods used for the development of SARS-CoV vaccines during the past two decades, which include: viral vector-based vaccine, DNA vaccine, subunit vaccine, virus-like particles (VLPs)-based vaccine, inactivated whole-virus (IWV) vaccine and live attenuated vaccine.

In general, IWV vaccine is the most rapid approach for vaccine production following a new outbreak. However, the use of IWV as a vaccine in MERS was reported to be associated with hypersensitivity-type lung immunopathologic reaction in the mouse model (Agrawal et al., 2015), thereby limiting its potential. Subunit vaccine is by far the most popular method in the development of MERS vaccine, mostly focusing on the recombinant RBD of the S protein produced in heterologous expression systems. Subunit vaccines, however, are often administered along with adjuvants to boost the immunogenicity of the recombinant antigens. Nanoparticles such as VLPs-based vaccines are similar to subunit vaccines, in which only specific viral proteins are expressed. Unlike subunit vaccines, VLPs-based vaccines are comprised of recombinant viral proteins capable of self-assembling into larger particles resembling viruses. Although the immunogenicity of VLPs-based vaccines could be enhanced by adjuvants, the VLPs themselves can serve as adjuvants which increase the immunogenicity of displayed epitopes, particularly those of smaller ones (Murata et al., 2003; Quan et al., 2008). Live attenuated vaccines are composed of live viruses, which have been modified to remove or reduce their virulence. This type of vaccine is often very immunogenic, whereby a single administration without an adjuvant is sufficient to induce protective immunity. However, the risk of reversion to a virulent virus has limited its usage as MERS vaccine. Viral vector-based vaccine is one of the most popular approaches in developing MERS vaccines. Two out of the three candidate vaccines which have entered the clinical phase are viral vector vaccines. This approach utilizes well-studied virus replication system to display MERS-CoV antigen, thereby inducing protective immunity against MERS-CoV. Another candidate vaccine currently in phase I/II clinical trial is a DNA vaccine. Unlike other types of vaccines, DNA vaccine production does not involve virus replication, protein expression and purification, therefore reduce the cost of production. However, administration of DNA vaccines often requires an external device such as electroporator or gene gun, which eventually increases the cost of immunization. Table 2 summarizes the vaccine candidates against MERS-CoV infection, which are further discussed intensively in the following sections.

**TABLE 2:** Potential vaccine candidates against Middle East respiratory syndrome-coronavirus.

**Vaccine type**	**Vector and antigen**	**Administration route**	**Results**	**References**
Viral-vector	rAd5 encoding S1 protein	IM	Immunization with rAd5 constructs expressing CD40-targeted S1 fusion protein (rAd5-S1/F/CD40L) offered complete protection to hDPP4 transgenic mice against MERS-CoV challenge and prevented pulmonary perivascular hemorrhage.	Hashem et al., 2019
	rAd5 or rAd41 encoding S protein	IM or IG	IG administration of rAd5-S or rAd41-S elicited antigen-specific IgG and neutralizing antibody in serum, but T-cell responses were not detected. A single IM injection of Ad5-S or Ad41-S induced systemic humoral response in addition to the functional antigen-specific T-cell responses in the spleen and pulmonary lymphocytes of the mice, which persisted for several months.	Guo et al., 2015
	rAd5 encoding S protein or S1	IM and later boosted with IN	Immunized mice demonstrated antibody responses against spike protein, which neutralized MERS-CoV *in vitro*. Stronger neutralizing antibody responses were observed in the mice vaccinated with the vector encoding the shorter S1 protein than the full-length S protein.	Kim et al., 2014
	ChAdOx1 encoding S protein^*^	IM or IN	Single dose intranasal or intramuscular immunization protected transgenic BALB/c mice against lethal virus challenge. Immunogenicity and efficacy were comparable between immunization routes.	Munster et al., 2017
	ChAdOx1 encoding S protein^*^	IM	Single dose immunization with ChAdOx1 MERS vaccine with tPA induced 5 logs of neutralizing antibodies in BALB/c mice.	Alharbi et al., 2017
	MVA encoding S protein	IM	Immunization with MVA MERS vaccine containing tPA regulated by F11 promoter induced 4.7 logs of neutralizing antibodies in BALB/c mice.	Alharbi et al., 2017
	MVA encoding S protein^∗∗^	IM or SC	Both immunization routes induced neutralizing antibodies and CD8+ T-cell responses in mice. Vaccinated mice were protected against MERS-CoV challenge infection after transduction with the hDPP4 receptor.	Volz et al., 2015
	MVA encoding S protein^∗∗^	IM	Neutralizing antibody responses were induced in immunized mice.	Song et al., 2013
	MVA encoding the N-protein	IM or IP	CD8+ T-cell response was elicited in the immunized mice in both immunization routes.	Veit et al., 2018
	NDV encoding S protein	IM	Recombinant NDV expressing MERS-CoV S protein induced neutralizing antibodies in BALB/c mice and Bactrian camels.	Liu et al., 2017
Viral-vector and nanoparticle	rAd5 and MERS-CoV S nanoparticle	IM	Heterologous prime-boost vaccination with rAd5-S protein and alum-adjuvanted recombinant S protein induced both Th1 and Th2 immune responses in SPF BALB/c mice.	Jung et al., 2018
DNA	DNA encoding S protein^∗∗∗^	IM followed by EP	The DNA vaccine was immunogenic in mice, camels and rhesus macaques. When the immunized macaques were challenged with MERS-CoV, characteristic clinical symptoms including pneumonia were reduced.	Muthumani et al., 2015
	DNA encoding S or S1 protein	IM	DNA encoding S1 protein elicited stronger antibody and cellular immune responses in mice than that encoding the S protein. Both DNAs encoding S1 and S proteins induced neutralizing antibodies that cross-reacted with MERS-CoV strains of human and camel origins.	Al-Amri et al., 2017
	DNA encoding S1 protein	IM	Immunization with DNA encoding S1 protein and passive transfer of immune sera from the vaccinated mice protected hDPP4-transduced-mice from MERS-CoV infection.	Chi et al., 2017
Subunit	MERS-CoV S1 protein	SC	Adjuvanted (MF59) MERS-CoV S1 protein protected hDPP4 transgenic mice against lethal MERS-CoV challenge, where the protection correlated well with the neutralizing antibody titer.	Wang et al., 2017c
	MERS-CoV S1 protein	IM	Immunization with adjuvanated (Advax HCXL adjuvant and Sigma Adjuvant System) S1 protein reduced and delayed virus shedding in the upper respiratory tract of dromedary camels and provided complete protection in alpaca against MERS-CoV challenge.	Adney et al., 2019
	MERS-CoV S protein trimer on Fd	IM	Recombinant prefusion trimeric MERS-CoV S protein induced high titer of neutralizing antibodies in BALB/cJ mice.	Pallesen et al., 2017
	RBD trimer on Fd	SC or IM	Adjuvanted (alum) RBD-Fd induced neutralizing antibodies in BALB/c mice and protected (83%) hDPP4 transgenic mice against lethal MERS-CoV challenge.	Tai et al., 2016
	RBD fused to Fc	SC	Adjuvanted RBD-Fc induced high titer of neutralizing antibodies in BALB/c mice and New Zealand white rabbits.	Ma et al., 2014b
	RBD fused to Fc	SC	Mice immunized with the vaccine and Montanide ISA 51 adjuvant produced neutralizing antibodies which inhibited binding of the RBD to DPP4 receptor, neutralizing MERS-CoV infection.	Du et al., 2013
	RBD fused to Fc	IN or SC	Mice vaccinated with both immunization routes in the presence of adjuvants (Montanide ISA 51 adjuvant for SC and Poly(I:C) for IN) elicited systemic humoral immune responses. Stronger systemic cellular immune responses and local mucosal immune responses were observed in mice immunized via IN route.	Ma et al., 2014a
	RBD fused to Fc	IM	hCD26/DPP4 transgenic mice immunized with the vaccine in the presence of adjuvant, AddaVax elicited neutralizing antibodies and were protected against MERS-CoV infection.	Nyon et al., 2018
	RBD fused to Fc	SC	Mice immunized with the vaccine alone produced detectable neutralizing antibodies and cellular immune responses. Immunogenicity of the vaccine improved when the adjuvants such as Freund’s adjuvant, alum, monophosphoryl lipid A, Montanide ISA51 or MF59 was included in the formulation. MF59 was demonstrated to be superior in enhancing the vaccine immunogenicity and protection against viral challenge.	Zhang et al., 2016
	Recombinant RBD	IM or SC	When the subunit vaccine was administered together with combination of alum and CpG ODN, optimized RBD-specific humoral and cellular immunity were elicted. Robust RBD-specific antibody and T-cell responses were induced in mice immunized with the vaccine in combination with IFA and CpG ODN, but low level of neutralizing antibodies were induced.	Lan et al., 2014
	Recombinant RBD	IM	Rhesus macaques immunized with the subunit vaccine and alum adjuvant produced neutralizing antibodies and experienced mitigated clinical symptoms when challenged with MERS-CoV.	Lan et al., 2015
	rNTD of S protein	IM	Immunization with rNTD of MERS-CoV S protein adjuvanted with alum induced neutralizing antibodies and reduced the respiratory tract pathology of BALB/c mice challenged with MERS-CoV.	Jiaming et al., 2017
VLPs	MERS-CoV VLPs	IM	Co-administration of the VLPs-based vaccine and alum activated RBD specific humoral and cellular immune responses in rhesus macaques.	Wang et al., 2017b
	S protein nanoparticles	IM	S protein produced in the baculovirus insect cells expression system assembled into nanoparticles of approximately 25 nm. Mice immunized with these nanoparticles in the presence of alum produced high titer of neutralizing antibody.	Coleman et al., 2014
	S protein nanoparticles	IM	The vaccine together with Matrix M1 adjuvant activated S protein specific humoral immune responses, and protected hDPP4 transduced mice against viral challenge.	Coleman et al., 2017
	CPV VLP displaying RBD	IM	Immunization of the mice with the chimeric VLPs displaying RBD in the presence of adjuvants [alum or Poly(I:C)] elicited neutralizing antibody responses as well as cellular immune responses.	Wang et al., 2017a
	Influenza A VLP displaying S protein	IM	Immunization of the mice with the chimeric VLPs displaying RBD in the presence of a combination of adjuvants (alum and CpG ODN) elicited neutralizing antibody responses.	Lan et al., 2018
Nanoparticle	Ferritin displaying RBD	IM	Immunization with chaperna-mediated ferritin nanoparticle displaying MERS-CoV RBD adjuvanted with MF59 induced RBD-specific antibodies in BALB/c mice which inhibited RBD binding to hDPP4 receptor protein.	Kim et al., 2018
Inactivated whole -virus	MERS-CoV	IM	Mice immunized with the inactivated vaccine in the presence of a combination of adjuvants (alum and CpG ODN) elicited neutralizing activity but not cell-mediated immunity. This vaccine also protected hDPP4 transduced mice against MERS-CoV challenge.	Deng et al., 2018
	MERS-CoV	IM	Gamma radiation-inactivated MERS-CoV induced neutralizing antibodies and reduced viral load in hDPP4 transgenic mice but may cause hypersensitivity-type lung immunopathologic reaction upon MERS-CoV challenge.	Agrawal et al., 2016
	Chimeric RABV displaying S1 protein	IM	The inactivated vaccine induced high titer of neutralizing antibodies in mice, and protected hDPP4-transduced-mice against MERS-CoV infection.	Wirblich et al., 2017
Live-attenuated	MERS-CoV mutant	-	The mutant was produced by deleting the *E* gene of the MERS-CoV. This mutant lacked infectivity but was single-cycle replicative.	Almazan et al., 2013
	MERS-CoV mutant	IN	Attenuated MERS-CoV through mutation of NSP16 (D130A) protected CRISPR-Cas9- targeted 288–330^+/+^ C57BL/6 mice from mouse-adapted MERS-CoV challenge.	Menachery et al., 2017
	MV expressing full-length or truncated, soluble variant of S protein	IP	The recombinant MV was replication competent. Immunization of the type I interferon receptor-deficient (IFNAR^–/–^) CD46Ge mice with the recombinant MV induced both MV and S protein specific neutralizing antibodies as well as cellular immune responses. The recombinant MV protected hDPP4-transduced-mice against viral challenge.	Malczyk et al., 2015
	MV expressing N protein	IP	Recombinant MV expressing MERS-CoV N protein induced N-specific T cell responses in IFNAR^–/–^-CD46Ge mice.	Bodmer et al., 2018
	Recombinant VSV expressing S protein	IN or IM	Recombinant VSV was produced by replacing the glycoprotein of VSV with the S protein of MERS-CoV. The recombinant virus induced neutralizing antibodies and T cell responses in rhesus macaques after a single intramuscular or intranasal immunization dose.	Liu et al., 2018

MERS-CoV, Middle East respiratory syndrome-coronavirus; rAd5, recombinant human adenovirus type-5; rAd41, recombinant human adenovirus type-41; MVA, modified vaccinia virus Ankara; ChAdOx1, chimpanzee adenovirus, Oxford University #1; NDV, Newcastle disease virus; MV, measles virus; CPV, canine parvovirus; RABV, rabies virus; VLP, virus-like particle; NSP, non-structural protein; S protein, spike protein; S1 protein, spike protein receptor binding subunit; RBD, receptor-binding domain in S1; N protein, nucleocapsid protein; rNTD, recombinant N-terminal domain; Fd, foldon trimerization motif; Fc, Fc region of human IgG; tPA, leader sequence of the human tissue plasminogen activator gene; IFNAR^–/–^-CD46Ge mice, genetically modified mice deficient of type I IFN receptor and transgenically expressing human CD46; SPF, specific-pathogen-free; IM, intramuscular; IN, intranasal; IP, intraperitoneal; SC, subcutaneous; IG, intragastric; EP, electroporation. Vectors and antigens marked with “^*^” have entered phase I clinical trial (^*^MERS001; ^∗∗^MVA-MERS-S; ^∗∗∗^GLS-5300).

## Viral Vector-Based Vaccine

The first viral vector-based vaccine was reported by Moss et al. (1984) who developed a potential hepatitis B vaccine using the vaccinia viral vector. Unlike subunit or inactivated vaccines, which generally function as extracellular antigens, a viral vector works by carrying a DNA encoding immunogenic components into host cells, followed by intracellular antigen expression, thereby activating a broad spectrum cell-mediated immunity in addition to the humoral immune responses. Majority of the viral-vector based vaccines do not require adjuvant for optimum efficacy (Ura et al., 2014). Adenovirus and modified vaccinia virus Ankara (MVA) are the two most common viral vectors used in the development of MERS-CoV vaccines.

Mice immunized intramuscularly with the recombinant human adenoviral (type 5 or 41) vector encoding the full-length S protein were shown to induce systemic neutralizing antibodies and mucosal T-cells immunity. Intriguingly, no mucosal T-cell response was detected when the vaccine was administered via an intragastric route, contrary to previous findings which suggested the importance of mucosal vaccination in activating the mucosal immunity (Guo et al., 2015). A recombinant human adenovirus type 5 (rAd5) vector encoding the shorter S1 extracellular domain of the S protein was reported to elicit slightly stronger neutralizing antibody responses than that encoding the full-length, suggesting the effect of immunofocusing (Kim et al., 2014). A recent study by Hashem et al. (2019) demonstrated that rAd5 constructs expressing CD40-targeted S1 fusion protein (rAd5-S1/F/CD40L) offered a complete protection to hDPP4 transgenic mice against MERS-CoV challenge, and prevented pulmonary perivascular hemorrhage. Additionally, Jung et al. (2018) showed that heterologous prime-boost vaccination with rAd5-S protein and alum-adjuvanted recombinant S protein nanoparticle successfully induced both the Th1 and Th2 immune responses in specific-pathogen-free BALB/c mice.

Pre-existing immunity against human adenovirus in human population is widespread, hampering its clinical application as a vector for vaccine development (Fausther-Bovendo and Kobinger, 2014). Recent developments of new adenovirus vectors for vaccine antigen delivery focus on the serotype to which human population is less exposed. Chimpanzee adenovirus (ChAdOx1) represents an attractive alternative to the human adenoviral vector due to its good safety profile and lack of pre-existing immunity in human population (Dicks et al., 2012), and has since been employed in the vaccine development against MERS-CoV infection. The recombinant ChAdOx1 encoding full-length S protein (ChAdOx1 MERS) was shown to be immunogenic in mice, and lethal virus challenge using hDPP4 transgenic mouse model further demonstrated its high protective efficacy against MERS-CoV (Alharbi et al., 2017; Munster et al., 2017). It is noteworthy that the immunogenicity of S protein could be improved by insertion of a gene encoding the signal peptide of human tissue plasminogen activator (tPA) upstream of the *S* gene of MERS-CoV, in both ChAdOx1 and MVA vectors (Alharbi et al., 2017). Currently, a candidate MERS-CoV vaccine known as MERS001, which contains the ChAdOx1 encoding the S protein of MERS-CoV is at phase I clinical trial. The trial is estimated to be completed by December 2019, in which the safety and immunogenicity of MERS001 at different dosage are being studied in healthy adult volunteers recruited and sponsored by the University of Oxford, United Kingdom (National Institutes of Health [NIH], 2018b).

Recombinant MVA encoding the full-length S protein represents another potential MERS-CoV vaccine candidate due to its good safety profile, decent immunogenicity, and high protective efficacy against MERS-CoV (Song et al., 2013; Volz et al., 2015; Alharbi et al., 2017). Another candidate vaccine currently in phase I clinical trial is MVA-MERS-S. The trial is being performed by the University Medical Center Hamburg-Eppendorf, Germany, in which the safety and immunogenicity of MVA-MERS-S in healthy adult volunteers are being assessed (National Institutes of Health [NIH], 2018c). Apart from the S protein, the highly conserved N protein of MERS-CoV was inserted into MVA, and inoculated into mice. Although the recombinant MVA encoding the N-protein elicited CD8+ T-cell response in the immunized mice, its protective efficacy was not investigated (Veit et al., 2018).

Apart from adenovirus and MVA, Newcastle disease virus (NDV) was also used as a viral-vector for displaying MERS-CoV S protein. The NDV-based vaccine candidate induced neutralizing antibodies in BALB/c mice and Bactrian camels (Liu et al., 2017). Although viral vector-based vaccines are able to induce robust immune responses, they are not free from drawbacks, which include pre-existing immunity against viral vector, risk of pathogenesis, low viral titer production, and potential tumorigenesis (Ura et al., 2014).

## DNA Vaccine

DNA vaccine is composed of a recombinant plasmid encoding immunogens. This vaccine is typically delivered via direct injection, gene gun, or electroporation into host cells, where the immunogens can be expressed and prime the immune system (Ferraro et al., 2011). DNA vaccine offers two distinct advantages over the subunit or protein-based vaccine: the ease of DNA manipulation and low cost of production (Leitner et al., 1999).

Similarly, all DNA vaccines developed against MERS-CoV target the S protein or the shorter S1 domain of MERS-CoV. DNA encoding the full-length S protein was shown to induce neutralizing antibodies and robust cell-mediated immunity in mice, macaques, and camels. When the immunized macaques were challenged with MERS-CoV, characteristic clinical symptoms including pneumonia were mitigated (Muthumani et al., 2015). GLS-5300 is one of the three candidate vaccines currently in a clinical trial. Sponsored by the GeneOne Life Science, Inc., Korea, a phase I clinical trial to test the vaccine’s safety profile in human volunteers was completed in the Walter Reed Army Institute of Research, United States (National Institutes of Health [NIH], 2016). Currently, the phase I and phase II clinical trials are being performed in the International Vaccine Institute, Korea, to further evaluate the safety and immunogenicity of GLS-5300, as well as a device for electroporation (CELLECTRA ^>^ 2000 Electroporation) (National Institutes of Health [NIH], 2018a).

To avoid the possible adverse effects induced by the full-length S protein, other researchers revealed that immunization with a DNA encoding the S1 domain, and passive transfer of immune sera from the vaccinated mice protected hDPP4-transduced-mice from MERS-CoV infection (Chi et al., 2017). The DNA encoding the S1 domain was also demonstrated to be more superior than that encoding the full-length S protein in eliciting antibody and cellular responses. Both DNAs encoding the S1 and S proteins were shown to induce neutralizing antibodies that cross-reacted with MERS-CoV strains of human and camel origins (Al-Amri et al., 2017). Despite the effectiveness of DNA vaccines, spontaneous plasmid integration into host genomes represents a potential risk, but the probability is extremely low (Ledwith et al., 2000).

## Subunit Vaccine

In general, subunit vaccines have the highest safety profile among all current vaccines despite their low immunogenicities (Du et al., 2016b). Precautions should be taken during the development of MERS-CoV vaccines based on the S protein to avoid induction of non-neutralizing antibodies. Unlike the full-length S protein, RBD of MERS-CoV comprises the critical neutralizing domains but lacking the non-neutralizing immunodominant region. Therefore, upon immunization, the RBD-based vaccines are restricted to produce RBD-specific neutralizing immune responses, thus are incapable of inducing non-neutralizing antibodies that may potentially contribute to harmful pathological effects (Du and Jiang, 2015; Wang et al., 2015a). From the safety and effectiveness perspectives, the RBD is a more promising candidate in the development of MERS-CoV vaccines over the full-length S protein.

The RBD of MERS-CoV was reported to induce neutralizing antibodies against multiple strains of MERS-CoV due to the presence of several conformational neutralizing epitopes (Du et al., 2016b). Any MERS-CoV strains with a single mutation in an epitope may not suffice to escape the RBD-specific neutralizing antibodies. Wang et al. (2015a) demonstrated that an amino acid mutation at position 509 (aspartic acid to glycine substitution) in RBD rendered the mutated strain resisted to neutralization by a RBD-specific monoclonal antibody, F11, but susceptible to another RBD-specific monoclonal antibody, D12. Both of these antibodies could bind to different regions of the RBD of MERS-CoV. Similarly, the RBD of SARS-CoV also consists of multiple neutralizing domains that are capable of inducing broad neutralizing immune responses against many SARS-CoV strains (He et al., 2006). Development of antibody escape mutants may require a mutation in two or more epitopes in the RBD of MERS-CoV, which is less likely to take place, and if developed, may exhibit reduced viral fitness (Tang et al., 2014; Tai et al., 2017).

It was demonstrated that the MERS-CoV S1 protein with MF59 adjuvant protected hDPP4 transgenic mice against lethal MERS-CoV challenge, where the protection correlated well with the neutralizing antibody titer (Wang et al., 2017c). In addition, adjuvanted recombinant S1 proteins (Advax HCXL adjuvant and Sigman Adjuvant System) reduced and delayed virus shedding in the upper respiratory tract of dromedary camels (MERS-CoV animal reservoir), and provided complete protection in alpaca (a surrogate infection model) against MERS-CoV challenge (Adney et al., 2019).

In general, MERS-CoV subunit vaccines based on the S1 domain require the use of adjuvant or fusion with an immune enhancer to heighten immunogenicity. Several studies have indicated that RBD fused with Fc fragment of human IgG (RBD-Fc) elicited strong systemic neutralizing antibody and cellular immune responses in vaccinated mice (Du et al., 2013; Ma et al., 2014a; Tang et al., 2015; Nyon et al., 2018) and New Zealand white rabbits (Ma et al., 2014b). hDPP4-transduced-mice immunized with RBD-Fc were also protected from viral challenge (Ma et al., 2014a). Other adjuvants such as Freund’s adjuvant, alum, monophosphoryl lipid A, Montanide ISA51 and MF59 were also reported to further improve the immunogenicity and protection of RBD-Fc in mice, particularly MF59 is superior among these adjuvants (Zhang et al., 2016). In addition, co-administration of multiple adjuvants together with RBD antigen could synergistically improve the immunogenicity of the RBD-based subunit vaccine. Mice immunized with RBD antigen together with alum and CpG ODN produced stronger humoral and cellular immune responses than those immunized with RBD antigen and alum or CPG ODN alone (Lan et al., 2014). RBD-based subunit vaccine was also previously tested in the rhesus macaque model in the presence of alum. This vaccine formulation was shown to induce robust and sustained humoral and cellular immunities, and partially protected rhesus macaques from viral challenge (Lan et al., 2015).

As native spikes of MERS-CoV exist in the form of trimers, vaccine designs mimicking the native viral S proteins have also been reported (Tai et al., 2016; Pallesen et al., 2017). Through the use of foldon (Fd), a T4 fibritin trimerization domain, Pallesen et al. (2017) synthesized a recombinant prefusion trimeric MERS-CoV S protein, which induced high titer of neutralizing antibodies in BALB/cJ mice. Similarly, Tai et al. (2016) expressed RBD trimers on Fd, and demonstrated the vaccine’s protective efficacy (83% survival) in hDPP4 transgenic mice against lethal MERS-CoV challenge.

Although most of the subunit vaccine studies focused on the RBD of the S protein, a recent study by Jiaming et al. (2017) proposed the use of recombinant N-terminal domain (rNTD) of the S protein as another potential vaccine candidate. The rNTD, when used to immunize BALB/c mice, induced neutralizing antibodies and reduced the respiratory tract pathology of mice in a non-lethal MERS-CoV challenge.

Apart from focusing on the S protein, multivalent vaccines designed using *in silico* methods which contain the B cell and T cell epitopes of S, E, M, N and NSPs have been proposed (Srivastava et al., 2018). However, until now, no biological data have been presented for these multivalent vaccines. In addition, the N protein and S2 domain of S protein are more conserved among coronaviruses, representing other attractive targets in the development of a broad-spectrum coronavirus vaccine (Schindewolf and Menachery, 2019). Nevertheless, it is crucial to ensure that these proteins do not contribute to the ADE of MERS-CoV infection.

## Virus-Like Particles (VLPs)-Based Vaccine

Virus-like particles are nanoscale particles similar to the native viral particles but devoid of infectious genetic materials. They are composed of repetitive viral structural proteins with inherent self-assembly properties. VLPs are non-replicative and non-infectious. VLPs can be produced by expressing the viral structural proteins in a suitable expression system (Yong et al., 2015a, b; Ong et al., 2017). In general, VLPs-based vaccine is similar to the whole inactivated virus vaccine, but it does not require the viral inactivation step which may alter the antigenicity and immunogenicity of a viral protein. Because no live virus is involved in the manufacturing process, VLPs can be easily generated in a low-containment manufacturing environment (DeZure et al., 2016).

Virus-like particles of MERS-CoV were previously produced in baculoviral expression system by co-expressing the S, E and M proteins of MERS-CoV. The VLPs generated were indistinguishable from the authentic viral particle when observed under an electron microscope. These VLPs, when administered with alum induced neutralizing antibodies and a Th1-biased immunity in rhesus macaques (Wang et al., 2017b). Intriguingly, when the S protein of MERS-CoV was expressed alone, it self-assembles into nanoparticles of approximately 25 nm, about a quarter of the diameter of the authentic viral particle. Immunogenicity studies in mice demonstrated that these nanoparticles elicited antibody responses in the presence of alum, and when the adjuvant was replaced with Matrix M1 adjuvant, they induced a significantly higher titer of neutralizing antibodies (Coleman et al., 2014). Viral challenge in hDPP4-transduced-mice which had been immunized with Matrix M1 and S protein nanoparticles further proven the protective efficacy of this vaccine formulation against MERS-CoV (Coleman et al., 2017). As mentioned earlier under the viral vector-based vaccine, adjuvanted S protein nanoparticles as boosters in mice primed with rAd-5 S have also yielded promising Th1 and Th2 immune responses (Jung et al., 2018).

Advancement in genetic engineering enables VLPs to display different epitopes of viruses, producing chimeric VLPs (cVLPs) (Ong et al., 2017). Expression of the RBD of MERS-CoV fused to the VP2 structural protein of canine parvovirus (CPV) produced cVLPs displaying the RBD of MERS-CoV. These cVLPs were morphologically similar to native CPV and elicited both RBD-specific humoral and cell-mediated immunities in mice (Wang et al., 2017a). The cVLPs displaying the S protein of MERS-CoV and matrix 1 protein of influenza A virus were also developed, and shown to be immunogenic in mouse models. However, the actual protective efficacy of these cVLPs against MERS-CoV has yet to be investigated *in vivo* (Lan et al., 2018).

In addition to vaccines based on VLPs, non-viral nanoparticle such as ferritin has also been reported as a potential carrier for MERS-CoV antigen (Kim et al., 2018). Kim et al. (2018) utilized a chaperna-mediated ferritin nanoparticle to display MERS-CoV RBD. When adjuvanted with MF59, the ferritin-based nanoparticle induced RBD-specific antibodies in BALB/c mice, which inhibited RBD binding to hDPP4 receptor protein, suggesting its potential use as MERS-CoV antigen carrier (Kim et al., 2018).

## Inactivated Whole-Virus Vaccine

Inactivated whole-virus comprises the entire disease causing virion which is inactivated physically (heat) or chemically. IWV offers several advantages, including relatively low cost of production, good safety profile, and does not involve laborious genetic manipulation (DeZure et al., 2016). Nevertheless, production of IWV requires the live virus to be grown under a high-level containment, and the antigenicity of the immunogen could be altered in the viral inactivation step (DeZure et al., 2016).

Formaldehyde-inactivated-MERS-CoV induced neutralizing antibodies in mice, but not T-cell response. Supplementing this IWV with a combined adjuvant (alum and CpG ODN) was reported to enhance its protective immunity against MERS-CoV in mice transduced with hDPP4 (Deng et al., 2018). On the other hand, an inactivated bivalent whole virus vaccine that targets rabies virus (RABV) and MERS-CoV was recently developed using a recombinant vector encoding a fusion protein comprising the MERS-CoV S1 domain fused to the C-terminus of RABV G protein. Following expression, the S1 domain was incorporated into RABV particles (BNSP333-S1). When the mice were immunized with the chemically inactivated BNSP333-S1, robust neutralizing antibody responses against S1 and G proteins were detected. Inactivated BNSP333-S1 also protected hDPP4-transduced-mice against MERS-CoV challenge (Wirblich et al., 2017). Despite the benefits associated with IWV-based vaccines, inactivated MERS-CoV vaccine was reported to potentially cause a hypersensitivity-type lung immunopathologic reaction upon MERS-CoV challenge, even though it induced neutralizing antibodies and reduced the viral load in hDPP4 transgenic mice, similar to those observed in SARS-CoV (Agrawal et al., 2016).

## Live Attenuated Vaccine

Live attenuated vaccine is one of the most effective vaccines due to its capability to induce immunity similar to the natural infection. This vaccine contains viable but attenuated virus. Common approaches to develop a live attenuated vaccine include deletion of the viral genes that confer virulence, and via reverse genetic. In general, live attenuated vaccines are highly immunogenic, thus do not require adjuvant for optimal efficacy, and single immunization is usually sufficient to induce protective immunity. Nevertheless, live attenuated vaccines come with some unwanted limitations, particularly the risk of reversion to a virulent strain, and the absolute need for vaccine cold chain. Live attenuated vaccine is also not suitable for infants, immunocompromised individuals, and elderly people (Lauring et al., 2010).

A live attenuated vaccine against MERS-CoV was previously developed by deleting the *E* gene of MERS-CoV (rMERS-CoV-ΔE). This engineered virus lacked infectivity and replicated in a single cycle. Vaccines based on the live attenuated viruses could pose biosafety problems associated with the risk of virulence reversion, whereas rMERS-CoV-ΔE is propagation defective in the absence of E protein, preventing a straightforward reversion to virulence, thus providing a safer alternative (Almazan et al., 2013). More recently, a live attenuated MERS-CoV was generated through mutation of NSP16 (D130A), where the attenuated virus protected CRISPR-Cas9-targeted 288–330^+/+^ C57BL/6 mice from a mouse-adapted MERS-CoV challenge (Menachery et al., 2017).

Other than MERS-CoV, a replication competent recombinant measles virus (MV) was used as a platform for the development of live attenuated MERS-CoV vaccine. The recombinant MV was engineered to express the full-length S protein (MV_*vac*__2_-CoV-S) or its truncated version (MV_*vac*__2_-CoV-solS). Both MV_*vac*__2_-CoV-S and MV_*vac*__2_-CoV-solS were shown to induce neutralizing antibodies and cell-mediated immune responses against MV and MERS-CoV, and protected hDPP4-transduced-mice from MERS-CoV challenge (Malczyk et al., 2015). Three years later, Bodmer et al. (2018) compared the MV_*vac*__2_-CoV-S with its UV-inactivated derivative, and showed that the inactivated version did not induce any specific immune response against both the MV and MERS-CoV. Concurrently, Bodmer et al. (2018) constructed a live attenuated recombinant MV expressing MERS-CoV N protein (MV_*vac*__2_-MERS-N), and its administration into IFNAR^–/–^-CD46Ge mice (genetically modified mice deficient of type I IFN receptor and transgenically expressing human CD46) induced N-specific T cell responses, although not as strong as those of MV_*vac*__2_-CoV-S. Similarly, in another study, a viable recombinant vesicular stomatitis virus (VSV) with its G protein replaced with the S protein of MERS-CoV also elicited both humoral and cell-mediated immunities in rhesus macaques (Liu et al., 2018).

## Obstacles in Bringing Mers Vaccines to the Market

Development of MERS vaccines started immediately following the discovery of MERS-CoV in 2012. Pre-clinical trials on animal models capable of recapitulating the clinical signs and symptoms in the human are a must prior to clinical trials and licensing of a vaccine (Gerdts et al., 2007). The choice of an animal model is generally preferable to be as phylogenetically closer as possible to the human (Swearengen, 2018). Therefore, majority of the vaccine candidates will be evaluated in non-human primates such as chimpanzees, rhesus macaques (Sibal and Samson, 2001) or marmosets (Carrion and Patterson, 2012). Employing these animal models in experiments, however, is extremely costly (Gerdts et al., 2015). Before involving non-human primates in a vaccine evaluation, strong justification or supporting evidence from *in vitro* analysis, or more preferable from animal studies such as small rodents are often required (Gerdts et al., 2015). However, MERS-CoV cannot infect smaller rodents naturally, representing a huge challenge in initial vaccine developments (Goldstein and Weiss, 2017). Although transgenic mouse models for evaluation of MERS-CoV vaccines have been successfully developed, the costs of these transgenic animals are not affordable by many research groups, especially those from the less affluent parts of the world. This issue consequently delayed the development of an effective vaccine, and its advancement into clinical trial.

Funding is the primary drivers in any vaccine developments. Many vaccines demonstrating promising results at the pre-clinical stage require additional investments from the government or the private industry to advance into clinical trials (Hakoum et al., 2017). However, government funding for clinical trials is rather restricted, whereas private industry is generally profit oriented, of which the market size and potential profits are of priority (Smith, 2000). Unlike other widespread diseases such as hepatitis and influenza, MERS cases are primarily reported in Saudi Arabia apart from the Korea outbreak (Gossner et al., 2016). Its relatively low occurrence is likely to limit the market size of MERS vaccines, leading to lower interest by the private funding bodies. Although three potential MERS vaccine candidates have advanced into clinical trials, they are currently in phase I/II. As completing the entire trials often take 10 years and above, they are unlikely to be commercially available in the coming 3–5 years.

## Conclusion

Despite having a low occurrence of recorded human-to-human transmission, the recent MERS outbreak in South Korea which demonstrated virus emergence in second and third generation contacts has reignited public awareness regarding the danger of MERS-CoV. As no effective treatment against MERS is currently available, therefore the best solution is to develop a functional MERS vaccine to prevent MERS-CoV infection. Amongst the six types of vaccines discussed above, more studies are focused on the viral vector-based and subunit vaccines. Even though many promising vaccine candidates have been proposed and reported, as of now, only three potential MERS-CoV vaccine candidates have progressed to phase I clinical trials: a DNA vaccine (GLS-5300) and two viral vector-based vaccines (MVA-MERS-S and MERS001. It is still very likely that no MERS vaccine will be available in the market for human in the near future. Therefore, considerable efforts should be given to minimize delays in executing clinical trials, such as better understanding and coordination between sponsors, primary investigators, investigators, participants and stakeholders.

## Author Contributions

CY and HO wrote the manuscript. SY, KH, and WT reviewed, edited, and approved its final version.

## Conflict of Interest Statement

The authors declare that the research was conducted in the absence of any commercial or financial relationships that could be construed as a potential conflict of interest.
